# Shared decision-making with patients with limited health literacy – experiences and needs of GPs regarding values clarification

**DOI:** 10.1080/02813432.2025.2586416

**Published:** 2025-11-12

**Authors:** Laura Vriese, Bart Knottnerus, Nina Groenveld, Jany Rademakers, Trudy van der Weijden, Jesse Jansen

**Affiliations:** ^a^ Department of Family Medicine, Care and Public Health Research Institute (CAPHRI), Maastricht University, Maastricht, Netherlands; ^b^ Nivel–Netherlands Institute for Health Services Research, Utrecht, Netherlands

**Keywords:** Shared decision-making, values clarification, patient preference, health literacy, general practice

## Abstract

**Introduction:**

Values clarification, a key but under-implemented component of shared decision-making (SDM), involves identifying what matters to a patient relevant to a health decision. It is especially important for patients with limited health literacy (LHL), who often struggle to express preferences. General practitioners (GPs) play a central role in facilitating this process, yet their experiences are underexplored.

**Aim:**

To explore how GPs experience values clarification with patients with LHL, the challenges they face, and which support or strategies they consider helpful to better integrate values clarification into decision-making.

**Methods:**

We conducted semi-structured interviews with 15 GPs purposively selected from practices in lower socioeconomic areas. Interviews were audio-recorded, transcribed verbatim, and analyzed using the framework method.

**Results:**

Four themes emerged: GPs consider values clarification important but challenging; it goes hand in hand with problem analysis; trust and continuity of care are essential foundations; and relatives can support and hinder the process. GPs described two contrasting situations: patients with LHL adopting a passive role, prompting a more paternalistic approach, and patients with LHL with strong expectations that can conflict with clinical guidelines. GPs expressed needs for training, prompts or scripts, and strategies to explore expectations before consultations.

**Conclusion:**

Values clarification in the context of SDM with patients with LHL is complex and context dependent. Given their ongoing relationships with patients, GPs are well-positioned to facilitate this. GPs’ experiences indicate that values clarification may occur throughout the consultation - often intertwined with problem analysis - reflecting the dynamic nature of SDM in general practice.

## Introduction

Shared decision-making (SDM) is a collaborative process in which clinicians and patients discuss options, benefits, and harms, and the patient’s values, preferences, and circumstances to reach a decision [1]. While SDM has been widely promoted across healthcare domains, its application in general practice presents specific challenges. General practitioners (GPs) frequently face time constraints, must navigate multiple decisions, and encounter patients with a wide range of needs and backgrounds [2]. To add, compared with hospital settings, where most SDM models originate and where consultations often center on a single, protocol-driven decision [3], general practice involves more undifferentiated decisions and less structured encounters with patients presenting with a wide range of symptoms and questions [4].

These challenges are more pronounced in consultations with patients with limited health literacy (LHL). Health literacy encompasses an individual’s knowledge, motivation, and competence to access, understand, appraise, and apply health information [5]. Patients with LHL often struggle to process medical information and tend to adopt a more passive role during consultations [6–8], which limits their meaningful participation in SDM [9,10]. At the same time, SDM has been associated with positive outcomes in this group, including improved knowledge [11], empowerment [12], improved communication quality [13], and reduced decisional conflict [14], underlining the importance of involving all patients in decision-making.

A key goal of SDM is to ensure that decisions align with what matters to the patient. This involves values clarification: the process of identifying what matters to a patient relevant to a health decision [15]. Values clarification can be especially relevant for patients with LHL [16], as they often struggle with articulating their preferences [17]. Values clarification can improve alignment between decisions and patients’ values [11], thereby reducing risks of overtreatment or undertreatment [18], non-adherence to treatment plans [19], and decisional regret [20]. Despite its importance, values clarification is often inconsistently applied in clinical practice [21–23]. Many consultations focus primarily on conveying information about the different options and potential benefits and harms, while patients’ values remain implicit or unexplored [22]. As a result, SDM in practice may fall short of its intended goals. Therefore, a stronger focus on values clarification is advocated. GPs play a key role in facilitating SDM and values clarification [24], which requires skills such as active listening [24], showing empathy [25], and asking exploratory questions [26]. However, various barriers may hinder values clarification, including limited consultation time [27] and the communication challenges associated with LHL (e.g. passive attitude and difficulty with articulating preferences [8,17]). Conversely, facilitators for values clarification may include a trusting GP–patient relationship [28–30] and support from relatives [31,32].

To address the complexity of SDM with patients with LHL in general practice, adaptations to existing SDM models have been suggested. For example, including a preparatory step to ensure that patients understand their diagnosis, an evaluation step to review the decision (process) [33], visual risk communication [34], and integration of the teach-back method (where patients summarize key information in their own words) to enhance understanding and engagement [35]. While these adaptations aim to improve overall SDM, specific strategies for values clarification for patients with LHL are still lacking. Existing values clarification methods [15,27,36–38] are often unsuitable for this group because they exceed recommended complexity levels [39,40], and research on how values clarification is practiced with patients with LHL, particularly in general practice, is sparse. Our previous study [41] showed that SDM and values clarification were applied rarely with patients with LHL in general practice. The current study, therefore, aims to explore how GPs experience values clarification with patients with LHL, the challenges they face, and which support or strategies they consider helpful to better integrate this process into general practice.

## Materials and methods

### Study design

This qualitative interview study explored GPs’ experiences and needs regarding values clarification with patients with LHL. All participating GPs had previously taken part in a related study where their consultations with patients with LHL and multimorbidity were video-recorded [41]. In that study, SDM was assessed using OPTION^5^, SDM-Q-9, and SDM-Q-Doc, and a qualitative analysis to describe how values clarification occurred. The overarching aim of these studies is to improve SDM, particularly values clarification, with patients with LHL. GPs were aware of these studies being part of a larger project, but the findings of the observational study were not shared with GPs during the interviews to encourage openness and a safe environment. We followed the COREQ reporting guideline [42]. Ethical approval was granted by the Research Ethics Committee of Maastricht University (FHML-REC/2022/094).

### Participants

GPs who participated in the previous study were asked to participate (*n* = 20). These GPs were purposively selected GPs from practices in lower socioeconomic status areas (based on postal code [43]) in the Netherlands. GPs in training were also allowed to participate if they were in their final year of training. Participating GPs received €75 as compensation.

### Procedure

Interviews were conducted by LV or Master of Medicine student NG, using a semi-structured interview guide (Appendix A) developed by the research team (LV, BK, JR, TvdW, and JJ), based on our previous study and literature on SDM and values clarification. Topics included GPs’ experiences with patients with LHL (perceived prevalence, subgroups, and methods for assessing health literacy), strategies, barriers, facilitators, key situations for values clarification, and their support needs (e.g. tools or training). We also asked whether support should target GPs, patients, or both. The guide was pilot-tested with a GP in training, leading to minor changes in wording. This data was not included in the analysis.

Before the interview, GPs provided written consent and updated previously recorded demographic data (see Table 1). Interviews took place via video call (Microsoft Teams). We encouraged GPs to share real-life examples. All interviews were audio-recorded, transcribed verbatim, and anonymized. During the process, emerging themes were discussed with the research team. After interviewing 13 GPs, no new themes emerged, and the final two interviews (14–15) confirmed that data saturation had been reached.

### Data analysis

Interviews were analyzed using the Framework method, a structured approach within the broader family of analytical approaches (such as e.g. thematic and content analysis) that follows a predefined set of steps and organizes data systematically to facilitate coding and theme development and comparison within and across cases [45]. Analysis followed five steps: (1) Data familiarization through transcribing and openly coding the interviews line-by-line in ATLAS.ti 25.0.1 [46] (LV or NG); (2) Development of a thematic framework based on data-driven codes (LV, NG, TvdW, JJ); (3) Application and refinement of the framework, in which a second coder independently coded 10% of the interviews and in which discrepancies have been resolved in discussion (LV, NG); (4) Charting data (themes and quotes) in the framework (LV, NG); (5) Interpreting and summarizing data (LV, NG) and discussing this with research team (LV, BK, NG, JR, TvdW, JJ).

## Results

### GP and practice characteristics

Fifteen out of 20 invited GPs agreed to participate in the interview (75%). Of the remaining five, three had not consented to be approached for follow-up in our previous study, one declined due to lack of time, and one was unable to participate due to illness.

The average duration of the interviews was 44 min [range: 33–51 min]. The GP characteristics are displayed in Table 1. The GPs worked across the Netherlands and varied in age and experience. Most GPs reported a higher-than-average proportion of patients with LHL in their practice. GPs estimate patients’ health literacy based on use of language (e.g. proficiency), behavior (e.g. incorrect medication use or a passive attitude), and knowledge (e.g. misunderstandings), although they also noted that this is not always easy to assess.

**Table 1.** Demographic characteristics of GPs and their general practices.

Characteristics GPs (*n* = 15)	*n* (%)
Sex	
Male	5 (33%)
Female	10 (67%)
Age (years)	
Mean [range]	48.5 [29–65]
18–39 [*n* (%)]	3 (20%)
40–59	10 (67%)
60+	2 (13%)
Mean number of years working as GP [range]	16.2 [0–29]
Type of GP	
Practice owner	10 (67%)
Employed by a health center	2 (13%)
Locum GP	2 (13%)
GP in training (final year)	1 (7%)
Previous training in SDM	5 (33%)
Previous training in health literacy	2 (13%)
Characteristics general practices of the 15 GPs	*n* (%)
General practice type	
Solo practice	6 (40%)
Duo practice	3 (20%)
Group practice	4 (27%)
Health center	2 (13%)
Employed other physicians in practice	
Assistant practitioner	15 (100%)
Nursing specialist	6 (40%)
Physician assistant	1 (7%)
Multilingual care consultants	3 (20%)
Degree of urbanization of location of practice [44]	
Extremely urbanized	4 (27%)
Strongly urbanized	9 (60%)
Moderately urbanized	0 (0%)
Hardly urbanized	2 (13%)
Not urbanized	0 (0%)
SES-WOA score of location of practice [range]*	−0.252 [−0.567 − 0.102]

* Socioeconomic score of neighborhoods based on wealth, educational level and labor market participation (higher score reflects higher socioeconomic status. The average score in the Netherlands is about 0) [43].

### Themes regarding values clarification with patients with LHL

Four main themes emerged in the analysis: (1) GPs consider values clarification important but challenging, (2) Values clarification and problem analysis go hand in hand, (3) Trust and continuity of care as foundations for values clarification, and (4) Presence of a relative during consultations can support and hinder values clarification. Themes are also summarized in Table 2.

#### Theme 1: GPs consider values clarification important but challenging

GPs find values clarification important, especially for patients with LHL. GPs considered values clarification particularly relevant in decisions about palliative care or self-management treatment plans (e.g. physical exercises or taking medication), but less relevant in urgent or simple yes-or-no decisions (e.g. whether to do a blood test or whether to provide antibiotic cream). Some GPs reported feeling satisfied when successfully clarifying values, while many also expressed frustrations with the challenges involved.

*‘When patients have a strong preference for something I don’t really agree with. So then my own opinion about what the best treatment is clashes with what the patient wants. And that can be pretty frustrating.’* (GP1)

Challenges arise particularly in two contrasting situations: GPs described patients with LHL who may adopt a passive role in consultations, meaning patients were quiet, hesitant, or asked the GP to make decisions for them; and patients with strong prior expectations or specific requests, for example entering the consultation with expectations about getting a specific treatment or test, sometimes not in line with clinical guidelines. While many GPs spoke about certain patients who are generally more passive or demanding, they also noted that patient behavior can be context-dependent; for example, when patients had received information about medication or illness from others, they came to the consultation with stronger expectations. For both situations, GPs indicated that patients’ limited understanding of their situation, such as of the medical information or diagnosis, may contribute to their behavior, either making them adopt a passive role or leading them to present strong, sometimes inappropriate, expectations.

GPs explained that *patients who adopt a passive role* often struggle with understanding values clarification, making meaningful engagement difficult and leading them to defer decisions to the GP.

*‘And then I try to talk about it [values], but the response is often, “Well, but you’re the doctor, just do what’s best for me.”’* (GP4)

In response, many GPs admitted they often take a more paternalistic approach by providing clear directions, seeking consent after presenting a single option, or omitting alternatives rather than fully involving the patient in an open discussion about values.

‘*Well, shared decision-making, that’s not really the case in this situation. It’s more like: I say how it should be done and you’re going to do it, so to speak. […] You end up taking a more directive role rather than truly collaborating.’* (GP6)

‘*Of course, I don’t always discuss every possible option, but then there are options that I genuinely think aren’t suitable at that time. That’s different in my view. […] Otherwise, it would just overwhelm the patient*.’ (GP4)

In the second situation, where *patients may have strong prior expectations or specific requests*, GPs explained that patients frequently request or insist on unnecessary or even harmful tests or treatments, such as X-rays or antibiotics. GPs explained that these encounters often require time and negotiation to align perspectives, sometimes resulting in consciously making decisions that are not in line with evidence-based recommendations, to preserve the patient–GP relationship.

*‘I tell the patient, ‘I’ll refer you this time, but I can already tell you it won’t help. Then we’ll see each other again afterwards and we’ll make a new plan.’ That way, I build trust, like, ‘See, I was right.’ Over time, they start to believe in what I’m saying. Yeah, that’s called building a doctor–patient relationship. It’s a give-and-take, and sometimes I give a little just to earn some trust.*’ (GP9)

This can lead to internal conflict as most GPs also described feeling a strong ethical responsibility to avoid unnecessary or potentially harmful interventions.

*‘We get a lot of patients who want antibiotics when they’re sick, even when it’s clearly just a viral infection. That’s what they’re asking for. Uhm… yeah, I don’t go along with that. We won’t be doing that. So even though they clearly know what they want, I do think, well, in that case I’m the doctor.’* (GP4)

Many GPs explained that sometimes tension or disagreements can arise when patients do not understand or accept the GP’s reasoning behind a treatment advice, which some experience as frustrating. They indicated that this is relatively often the case in interactions with migrant patients, who may have different expectations of care.

*‘They [migrants with LHL] are just used to going straight to a specialist, not to a GP. They don’t really understand the whole system [with the GP being the one referring to specialized care] here. So, there you really have to… well, it takes extra time to explain it.’* (GP4)

#### Theme 2: Values clarification and problem analysis go hand in hand

More so than with patients with higher health literacy, GPs emphasize the importance of first reaching mutual understanding of the reason for their visit by exploring the health problem, before moving towards SDM about options. This preparatory step requires a different approach in the two situations that were described in Theme 1.

Many GPs reported that patients with LHL *who may adopt a passive role* often struggle to clearly articulate the reason for their visit. To address this, GPs stressed the importance of asking concrete, understandable questions to help reveal what truly concerns or matters to the patient.

*‘When you ask complicated questions like, “How do you think you’ll improve your health?” […] You just get blank stares. Yeah, that’s not really a question. Because that’s way too abstract. But if you ask more concrete things like, “What would help you feel better?” or “When do you feel good?”, that might work much better.’* (GP6)

*‘You can always ask, “What’s important to you?” […] “What’s your dream?” “How do you see your future?” And questions like that help uncover what really matters to them.*’ (GP15)

In contrast, GPs described that for patients with LHL *who may have strong prior expectations or specific request,* requests often mask underlying worries. It requires time and effort to explore the real reason behind the request, for example by addressing concerns and emotions.

*‘The question people come in with is often kind of hidden. People say things like, “I want a CT scan”, because they heard about it somewhere. Right, and then you go along with it and ask: “why?” Yeah, you really have to put in the time to figure out what it’s really about. Because often it’s not even the complaint itself, but they’re actually afraid of something. You need to uncover the real concern. […] That’s especially important with this group, I think.’* (GP13)

GPs differ in how they see the relationship between this preparatory step and values clarification. Many view it as a prerequisite for values clarification, since early exploration of the patient’s perspective and reason for the visit can create space for sharing values. Others, however, feel it can hinder meaningful discussions about values, because the effort required to clarify the patient’s health problem often takes up most of the consultation time, especially when the patient has multimorbidity, and does not necessarily provide insight into underlying values.

*‘Limited health literacy. Well, those patients often have multimorbidity too. […] Those often are more complex cases. […] It’s rarely something simple like, “I have a sore throat on the left side.” It’s vaguer, and it’s not just physical, there are lots of layers. So yes, it takes more time.’* (GP14)

To support patients with LHL, many GPs schedule additional follow-up appointments to stagger information provision and allow time for patients to reflect on their values.

#### Theme 3: Trust and continuity of care as foundations for values clarification

Most GPs emphasized that trust and continuity of care are essential for values clarification. While this applies to all patients, it is considered especially important for those with LHL, as these patients often struggle with expressing their concerns and what matters to them. Some GPs noted that when there is trust, patients are more likely to engage in values clarification.

*‘I think patients can only talk about what they want when they feel safe about what’s being said. They need to trust you first.*’ (GP2)

Some GPs explained that trust develops through continuity of care. Over time, continuity helps GPs develop a deeper understanding of the patient’s context and values, facilitating values clarification. Some GPs mentioned that a trusting, longstanding relation also helps them with values clarification as it makes it easier to interpret subtle cues (e.g. changes in behavior or tone) and to read between the lines.

*‘I’ve got lots of patients I’ve known for over twenty years. You get to know how someone ticks and what does or doesn’t suit them. At least, for a lot of them.’* (GP11)

As in the previous themes, the two situations also have a different impact here. Patients with LHL *who may adopt a passive role* often lack preconceptions about treatment options. Many GPs explained that these patients tend to accept the GP’s recommendations without much resistance, which GPs described as pleasant and convenient.

*‘You see, patients with stronger basic skills can think things through themselves and consider all the ifs and buts. But people with more limited abilities tend to think, “The doctor examined me, and said this”, and they basically trust that.’* (GP13)

Some GPs felt that, for *patients who may adopt a passive role*, values clarification can be misinterpreted as a sign of the GP being unsure, potentially undermining trust. In such situations, particularly when time is limited, GPs often feel compelled to take a more directive role.

Interviewer: *‘What do you do when someone says, “Well, you’re the doctor, aren’t you?”’*

GP: *‘That’s tricky, because that’s a sign the trust is fading. Then there’s this sort of tension in the consultation, which is unpleasant, because then you have to take up your role as the doctor again and just be a bit paternalistic.’* (GP13)

In contrast, some GPs described that it can be more challenging to build or maintain trust with patients with LHL *who may have strong prior expectations or specific requests*, especially when the GP’s clinical judgment conflicts with the patient’s wishes. In these cases, trust is perceived as fragile and only maintained if the GP fulfills the patient’s request, making values clarification more difficult.

*‘They [patients with LHL with strong prior expectations] sometimes come in with really strong ideas, like, “I need this,” or “This is what my body needs.” But it’s completely off. And sometimes, no explanation will change their mind.*’ (GP4)

As noted in Theme 1, some GPs occasionally comply with such requests to gain or maintain trust.

#### Theme 4: Presence of a relative during consultations can support and hinder values clarification

Some GPs noted that patients with LHL often bring someone to accompany them to consultations, generally a relative and sometimes a care professional (e.g. support worker) or translator. Many GPs mentioned that the presence of this person can either support or hinder values clarification, depending on their background and role. Relatives and care professionals or translators can enhance communication by helping patients to share their values, translating information, or supporting the patient after the consultation.

*‘Then it becomes more of a three-way conversation. You hope the person who came with them knows them well and can help make things clearer.’* (GP13)

*‘They often come with a support worker. That also makes it a bit easier, because they [the support worker] often write things down and say like, “We’ll talk about it again later and then we come back together.”’* (GP5)

In contrast, relatives who also have LHL themselves can make values clarification more challenging. They may struggle to understand medical information themselves or amplify distrust in the GP.

*‘But then sometimes the family doesn’t agree. Half of them is on board, the other half isn’t. […] But if you don’t trust me, or my judgment, you should not come here anymore.’* (GP11)

*‘Sometimes the family member gets caught up in the emotion and doesn’t understand either. […] Then I have to convince two people.’* (GP9)

Some GPs also raised concerns about relatives potentially influencing or misrepresenting the patient’s values. One GP described a situation in which a husband spoke on his wife’s behalf about wanting children, which raised doubts about whose wishes were truly being communicated.

*‘You don’t always know if you’re hearing the man’s wishes or the woman’s. It’s really important to make sure we’re acting on the woman’s wishes, not his.’* (GP15)

**Table 2.** Themes related to the two situations GPs identified.

	Situation 1: patient with LHL who may adopt a passive role	Situation 2: patient with LHL who may have strong prior expectations or specific requests
Challenge	The patient has difficulty with articulating the reason for the encounter expressing values/preferences understanding SDM and values clarification Leads to low engagement and deferring decisions to the GP	The patient requests unnecessary or potentially harmful interventions (e.g. X-rays, antibiotics) Can create tension with clinical judgement and clinical guidelines
Common underlying challenge	Patients often do not fully understand their diagnosis or situation	
GPs’ response	GPs often adopt a more paternalistic approach	GPs try to align perspectives, sometimes resulting in decisions that are not in line with evidence-based recommendations to preserve the patient-GP relationship
Focus of preparatory step	Ask concrete, understandable questions to uncover what truly matters to the patient	Explore the underlying reason behind the patient’s expectations or requests
Trust	GPs feel that Patients often accept GP advice due to lack of preconceptions Some patients interpret SDM and values clarification as uncertainty, which can undermine trust	GPs feel that trust is fragile and often depends on whether the GP meets patient expectations
Influence of a relative	The impact on values clarification depends more on the relative’s role: Helpful relatives (or care professionals or translators) can clarify values, translate, or provide emotional support Relatives with LHL may misinterpret information or foster distrust	

### GPs’ needs for support around values clarification with patients with LHL

Most GPs expressed a need for support in facilitating values clarification with patients with LHL. They emphasized that support should primarily target GPs, as patients should not be expected to lead this process. Some suggested a combined approach as values clarification is a shared effort. GPs identified three main strategies: (1) training in recognizing LHL and communicating with patients with LHL, (2) prompts or scripts for situations where values clarification is difficult, (3) gathering patient’s expectations and preferences before the consultation.

#### Training in recognizing and communicating with patients with LHL

GPs emphasized their interest in training focused on both recognizing LHL and communicating with these patients effectively. They especially valued general communication training, such as through simulated patient scenarios, and recommended its integration into medical education and accredited programs.

‘*Yeah, I do think it’s all about communication skills. Asking the right questions in the right way at the right time. You’ve got to invite the patient to really think along with you. Not just answer yes or no, but really consciously let them put into words what they feel, or where, or what they expect. I really think the conversation is super important for that.’* (GP14)

#### Prompts or scripts for situations where values clarification is difficult

Some GPs mentioned they would benefit from prompts and/or scripts (e.g. standardized, ready-to-use questions or explanations) to navigate conversations where values clarification is difficult. For *patients who adopt a passive role*, prompts or scripts can help to bring concerns or values to the surface. For *patients who have strong expectations or requests*, prompts or scripts can assist in explaining why certain requests may not be appropriate, while also helping to reveal the motivation behind the requests.

*You should have a few go-to phrases ready to use during consultations, that you can apply right away. It’s a really practical way to learn something, but it works for me. Just five sentences in your head that you can pull out when you notice it isn’t really working. So you can choose the right one for the right moment*.’ (GP8)

#### Gathering patients’ expectations and preferences before consultations

While many GPs try to allow extra time for values clarification through longer consultations, or additional follow-ups, they recognized this is not always feasible. To save time, a few suggested gathering patients’ concerns, expectations, and preferences before the consultation, *via* questionnaires or triage staff. Some GPs were already doing this and highlighted the value of well-trained assistants to assist in pre-consultation values clarification.

*‘My assistants always ask the question, “Why are you coming to the doctor?” and “What do you expect?” They’re good at it. They’ll also ask, “What do you think the doctor can do for you?” So my assistants, I think they’re pretty well trained in that.’* (GP10)

However, other GPs cautioned that pre-consultation questions may overwhelm patients or limit the GP’s ability to approach consultations with a ‘blank slate’, meaning without prior assumptions or expectations.

## Discussion

This study explored GPs’ experiences with values clarification in patients with LHL, the challenges they face, and perceived strategies to better integrate this process into practice. GPs considered values clarification important but often challenging with this group. They described spending much time understanding the patient’s health problem before initiating SDM. Trust and continuity of care were seen as essential for values clarification, acknowledging that trust is often fragile. Relatives were seen as both supporting and complicating values clarification, depending on the relative’s relationship with the patient and background. Two overarching and contrasting situations were described by GPs: on the one hand, patients with LHL may adopt a passive role, deferring decisions to the GP, prompting a more paternalistic approach. On the other hand, patients with LHL may have strong, sometimes unnecessary expectations, complicating values clarification as it creates tension between patient preferences and evidence based clinical care. These findings underscore the complexity of this process in general practice and the need for tailored strategies. GPs suggested three strategies: training focused on recognizing LHL and communicating with these patients effectively, prompts or scripts for situations where values clarification is difficult, and strategies to gather patient expectations and preferences before consultations.

For patients who adopt a passive role, many GPs in our study assumed that SDM would be too complex or overwhelming and responded with a more paternalistic approach. Some did not feel the need to involve these patients in the decision-making process, reflecting earlier findings that clinicians may default to directive communication styles based on perceived patient capability [47]. Although SDM is widely promoted as best practice, ambiguity persists about when and to what extent it is considered appropriate. Previous research has shown that even similar clinical scenarios have been interpreted differently, with some clinicians viewing them as suitable for SDM and others not, highlighting variation in how SDM is understood and applied across settings and professionals [48]. As a result, GPs may not always recognize a decision as one that warrants patient involvement.

Despite this uncertainty, most GPs emphasized that trust and continuity of care were essential for enabling values clarification, as they facilitate open, value-based conversations, consistent with earlier research [49]. Patients who adopt a passive role were often seen as expressing strong trust by readily accepting the GP’s judgement, a pattern also observed in the literature among patients with lower education levels [50]. However, while GPs interpreted this acceptance as a sign of trust, it remains unclear whether these patients are genuinely satisfied with a paternalistic approach. Research suggests that such passivity can also serve as a coping mechanism to manage decisional stress, rather than indicating actual trust or indifference [33,51–53]. In fact, many patients with LHL do want to be involved but face barriers, including limited understanding of SDM, low confidence, or feelings of disempowerment [33].

Some GPs also worried that initiating SDM, and particularly values clarification, might be perceived by patients with LHL as a sign of the GP being unsure, potentially undermining trust. This concern contradicts literature suggesting SDM builds trust [54], and may reflect varying or ambiguous understandings among clinicians and patients regarding the purpose and practice of SDM and values clarification in practice [48].

These findings underscore the risk that values clarification is omitted based on assumptions about patient needs, preferences, or capabilities and highlight the importance of always at least inviting patients to participate.

Conversely, when patients held strong expectations, GPs frequently encountered requests for interventions that were misaligned with clinical guidelines. Research shows that such expectations can hinder SDM [55,56], although effective values clarification can support negotiation towards mutually acceptable options [57]. GPs in our study attributed patients’ inappropriate requests to patients’ limited understanding of their medical situation, consistent with findings that mismatched expectations can result from patient’s limited comprehension of the situation [58]. Although GPs saw these requests as more frequent in patients with LHL, previous studies found no clear link with education level [59]. Still, patients with LHL are particularly susceptible to misinformation, which may distort their understanding of risks and benefits and thereby contribute to such requests [60]. Importantly, SDM presupposes the presence of clinical equipoise - that is, situations in which multiple medically justifiable options exist [48]. When patients insist on interventions that fall outside of evidence-based care, SDM in its conventional form may not be applicable. However, values clarification can still be relevant in such cases by uncovering the patient’s underlying concerns, beliefs, or unmet needs that give rise to the request. At the same time, patients’ assertiveness can facilitate engagement and communication, as recent research shows that assertive patient behavior can positively influence consultation outcomes by helping clinicians understand their preferences [61].

In situations where patients had strong expectations, GPs found that trust was more fragile: when they did not meet a patient’s expectation, trust could quickly erode, making values clarification more difficult. This aligns with research that low levels of trust hinder SDM, although trust can be rebuilt through open communication, empathy, and respect for autonomy [62].

Across both types of situations, whether patients adopted a passive role or held strong expectations, GPs identified patients’ limited understanding of their situation as the main barrier to effective values clarification. To uncover additional underlying barriers for values clarification, research from the patient’s perspective is needed.

Our findings invite reflection on for which patients and in which contexts SDM is the preferred approach [13,48], as GPs in our study described that some patients with LHL may not always wish to participate in decision-making. However, we recognize that this apparent unwillingness may reflect patients’ challenges in understanding their medical situation or engaging in SDM rather than a genuine preference to defer decisions. The two scenarios described by GPs - patients adopting a passive role or arriving with strong prior expectations for a medical intervention - reflect, respectively, paternalistic and consumer-oriented approaches [63], or the two extremes on the SDM continuum described by Kon [64]. While some GPs interpreted passivity as a lack of interest or willingness to engage, our findings suggest that for many patients with LHL, this apparent passivity reflects challenges with understanding information or their ability to participate. This aligns with research showing that most patients with LHL prefer to participate rather than defer decisions [33], though a smaller subset may genuinely prefer not to [65].

Recognizing this distinction is essential: patients who experience discomfort or apprehension regarding participation and prefer decision-making to their trusted clinician should not be forced into engagement [66]. Nevertheless, presuming a lack of interest solely on the basis of patient behavior may impede meaningful engagement and the provision of appropriate support [67]. Respecting autonomy, as Pilnick emphasizes, also includes acknowledging when patients prefer to trust clinicians to make decisions, while acknowledging their broader life context, challenges, and preferences [66]. This process of clarifying what matters to the patient is an iterative process as research shows that patients’ preferences can shift across contexts over the time [65]. Taken together, the application of SDM and values clarification in general practice requires flexibility, sensitivity, and tailoring to the diverse and changing needs, preferences, and capacities of patients.

GPs in our study emphasized the importance of a preparatory step to explore concerns, correct misunderstandings, debunk misinformation, and establish shared understanding before moving towards values clarification and SDM. This aligns with prior SDM research advocating a ‘step 0’ in consultations with patients with LHL to ensure clarity around diagnosis and context [33]. For patients who adopt a passive role, this step can encourage engagement; for patients with strong prior expectations, it can help uncover underlying motives. Some GPs felt that the time required for this initial step limited opportunities and time for more structured values clarification later in the consultation, leading them at times to either adopt a more directive approach or grant to patient requests that goes against the clinical guideline to preserve the GP-patient relationship. GPs described how this step precedes but also overlaps with values clarification, as clarifying concerns and expectations at the start of the consultation - as part of problem analysis - reveals what matters to patients and makes it an integral part of the values clarification process. These findings suggest that the boundaries between SDM, values clarification, and the preparatory step are not always clear-cut in clinical practice with patients with LHL. Rather than occurring as a clearly demarcated step in the SDM process, values clarification was described as an ongoing process, initiated at the start of the consultation. This is supported by research suggesting that values clarification can be integrated flexibly across different moments within or across consultations [68,69].

GPs identified three main needs around improving values clarification with patients with LHL. First, they emphasized training in recognizing LHL and improving communication skills. Our findings suggest that such training should also include a clearer understanding of when and how to apply SDM and values clarification, as GPs at times appeared to make premature or inaccurate assumptions about the suitability of SDM based on patient attitudes - such as interpreting passivity as a reason to adopt a more paternalistic approach [70]. In addition, since GPs considered patients’ limited understanding of their situation a barrier, training could address how to explain medical information in clear and accessible ways, so that patients are better able to participate with realistic expectations. Second, GPs expressed a need for simple, standardized prompts or scripts to support values clarification. These may help GPs feel more confident when navigating complex consultations, especially with patients who adopt a passive role or have strong prior expectations [71]. However, prompts alone are unlikely to be sufficient, as effective values clarification also requires GPs to be open to the patient’s story and respond with empathy and curiosity [72]. Combining the provision of such prompts or scripts with communication training appears particularly valued [73], highlighting the benefit of addressing these two strategies together. Third, opinions differed on gathering patient expectations and preferences before consultations; while some GPs in our study supported using triage staff or pre-consultation questionnaires, others preferred to start with a ‘blank slate’ (without prior assumptions or expectations). Given the known difficulties that patients with LHL may face with written materials, including questionnaires, such strategies require careful consideration to avoid increasing patient burden [5,74]. Moreover, a Cochrane review found that pre-consultation interventions offer limited benefits and may not reliably enhance communication or clinical outcomes [75]. One possible solution could be to schedule a separate, non-acute consultation with the GP or practice nurse to discuss values, similar to advance care planning for older adults [76]. A person-centered approach that includes a visual conversation tool outlining potential discussion topics is being developed to help patients with low socioeconomic status and chronic conditions in expressing what matters to them [77].

### Strengths and limitations

A strength of this study is that prior observational insights informed the researchers’ understanding, while keeping participants unaware of earlier findings to ensure openness. To our knowledge, this is one of the first studies specifically exploring the process of values clarification with patients with LHL in general practice. The participating GPs had extensive experience with this patient population, enabling them to provide multiple real-world examples. We also achieved data saturation, strengthening the reliability of our findings. Furthermore, our sample included GPs with varying years of experience and from diverse geographical locations, both urban and non-urban areas, as well as regions with higher and lower proportions of migrants, enhancing the transferability of our results.

A potential limitation is selection bias, as more engaged GPs may have participated. This underscores the need for improvement, since even engaged GPs reported significant barriers, likely even greater among less engaged peers. Another limitation is the possibility of socially desirable responses. We minimized this by ensuring anonymity and encouraging genuine experiences.

## Conclusion

This interview study highlights how GPs experience values clarification in the context of SDM with patients with LHL. Values clarification with patients with LHL is complex and context dependent. GPs described how the process often unfolds differently depending on whether patients adopt a passive role or have strong prior expectations or specific requests, yet in both situations, they identified the patients’ limited understanding of their situation as the main barrier to effective values clarification. GPs emphasized the importance of trust and continuity of care. GPs seem to have the ideal position for values clarification given their long-lasting relationship with patients and their relatives. Tailored strategies focusing on understanding the patient’s main concerns may help support GPs in integrating values clarification into everyday practice. GPs’ experiences suggest that values clarification may occur at multiple points in the consultation - often intertwined with the problem analysis - reflecting the flexible and dynamic nature of SDM in general practice.

### Implications

To support values clarification with patients with LHL in general practice, GPs could be actively involved in co-creating practical tools and approaches. Sufficient time is essential for applying values clarification. This may be achieved by spreading values clarification over multiple consultations, scheduling longer appointments, or offering separate, non-acute consultations focused on values. Communication training for GPs may also support values clarification with these patients. Finally, since GPs considered patients’ limited understanding of their situation a barrier to values clarification, such training could also focus on explaining medical information in a clear and accessible way, enabling patients to better understand, engage more actively, and develop well-informed expectations.

This is one of the first studies on the process of values clarification with patients with LHL in general practice. It is important to further substantiate and build on our results with research in a more diverse sample of GPs. In future research, a tailored intervention or tool could be designed to support values clarification for patients with LHL. However, many tools already exist that could be re-used or adapted. In all cases, it is important to test such tools with patients with LHL regarding feasibility and effectiveness. Importantly, before developing any intervention or tool for SDM and values clarification, experiences of patients with LHL should be thoroughly explored to ensure solutions align with their actual needs.

## Data Availability

The data that support the findings of this study are available from the corresponding author (LV) upon reasonable request.

## Appendix

### Appendix A. Interview guide

In the first part of our study, we visited GP practices and filmed consultations with patients who have limited health literacy and multimorbidity. We looked at how GPs make shared decisions with these patients. In this next phase of the research, we would like to explore your experiences with clarifying values with this patient group.

We are focusing on patients with limited health literacy, which are people who find it difficult to understand and apply health information, for example people with low educational levels, older adults, migrants, some of them have trouble reading or writing.

- Do you have many patients with limited health literacy in your practice?
  - Could you tell us a bit more about this group in your practice?
    - What ‘types’ of limited health literacy do you encounter most? (For example, older adults, migrants, people with lower education levels)
    - How do you assess or get a sense of a patient’s health literacy?
  - Do you adjust how you conduct the consultation for these patients?
    - Why or why not?
    - If yes, how do you adapt?
  We often hear from GPs that it can be challenging to clarify values with patients who have limited health literacy.
  - What has your experience been like?
    - Could you elaborate on that?
      - What are some examples of situations you’ve encountered?
        - When is it difficult?
          - In what kind of situations or with which types of patients?
          - Is this something you experience often?
        - Can you recall a specific case when it was hard to clarify values with a patient with LHL?
          - Could you elaborate on that? What made that situation challenging?
            - What obstacles did you encounter?
            - When it’s difficult to get a clear idea of a patient’s values, how do you handle it?
        - Can you also recall a time when it actually went smoothly?
          - What was different in that situation?
          - In what context, or with what type of patient, is it easier to clarify values?
        - Do you have other experiences or emotions you experience when trying to elicit and discuss patient values?
          - (Start without suggesting specific emotions) For example: frustration, irritation, exhaustion, discomfort, or perhaps relief or confidence?
          - In what situations or with what types of patients do you feel that way?
- (If not already addressed) Do you ever explicitly ask patients about their values? For example, their goals or whether they have a preference for or against a certain treatment?
- In your view, when is it especially important to clarify values in detail?
  - And when might it be less important?
- How do patients with limited health literacy usually respond when you ask about their values?
  - Could you give an example of how such a conversation has gone?
    - What did you do in that situation?
  - Are you usually able to uncover their values?
    - What happens when you succeed?
      - Is it more likely with certain types of patients?
- Do you use a particular method or approach to clarify values with patients who have limited health literacy?
  - Can you give an example of how that works in practice?
  - Who usually brings up the topic of values, you or the patient?
    - Would you prefer that to be different? Or do you think it works well this way?
- How do you take the patient’s values and priorities into account in your care decisions?
  - Can you give an example?
- What do you do when you’re not able to uncover the patient’s values?
- What would make it easier for you to clarify and discuss patient values?
  - What kind of support or resources would help?
    - For example: more training or experience, longer consultations, a tool, or a specific intervention?
      - Follow-up questions:
      - If training: What should that training cover?
      - If more time: What would an ideal consultation look like? How much more time, and how would you use it?
      - If a tool: What should be included in that tool?
      - If an intervention: Do you have any ideas for what that might look like?
  Our goal is to develop an intervention (this could be a training, a tool/product) to support better conversations about patient values, especially with patients who have limited health literacy.
- Should this intervention focus more on the GP or on the patient (or both)?
- Do you have ideas of what such an intervention could look like? (For example: training for GPs, a conversation aid for patients and doctors, or an informative video for patients)
  - Who or what would be needed to make this effective?
- Is there anything else you’d like to add, or anything you think we might have missed?
